# Supplementary material for: HLA Class II Polymorphism and Humoral Immunity Induced by the SARS-CoV-2 mRNA-1273 Vaccine
Source: Vaccines (Basel). 2022 Mar 6;10(3):402. doi: 10.3390/vaccines10030402 (PMC8949280; doi:10.3390/vaccines10030402)
Supplement: Supplementary file 1 [file vaccines-10-00402-s001.zip › Supplementary Table S1.pdf]

Supplementary Table S1. Quantification of specific IgG of protein S and HLA-class II typing of each participant.

Participant	BAU/mL	HLA-DRB1*	HLA-DRB1*	HLA-DQA1*	HLA-DQA1*	HLA-DQB1*	HLA-DQB1*
1	65	01:01	15:01	01:01	01:02	05:01	06:02
2	82	15:01	-	01:02	-	06:02	-
3	308	01:01	14:54	01:01	01:04	05:01	05:03
4	349	04:04	11:01	03:01	05:05	03:01	03:02
5	382	01:02	08:01	01:01	04:01	04:02	05:01
6	404	03:01	13:02	01:02	05:01	02:01	06:04
7	416	01:02	12:01	01:01	05:05	03:01	05:01
8	447	03:01	04:05	03:03	05:01	02:01	03:02
9	460	01:02	16:01	01:01	01:02	05:01	05:02
10	463	12:01	16:01	01:02	05:05	03:01	05:02
11	490	11:01	11:03	05:05	-	03:01	-
12	522	01:01	04:07	01:01	03:03	03:01	05:01
13	544	13:01	-	01:03	03:01	03:02	06:03
14	550	04:04	07:01	02:01	03:01	02:02	03:02
15	609	04:05	04:07	03:03	-	03:01	03:02
16	618	09:01	15:01	01:02	03:03	02:02	06:02
17	680	01:02	13:01	01:01	01:03	05:01	06:03
18	715	01:02	13:03	01:01	05:05	03:01	05:01
19	719	13:02	14:01	01:02	01:04	05:03	06:09
20	740	04:04	11:01	03:01	05:05	03:01	03:02
21	763	01:02	04:05	01:01	03:03	03:02	05:01
22	829	01:03	04:03	03:01	03:02	03:02	-
23	838	11:03	16:01	01:02	05:05	03:01	05:02
24	860	03:01	13:01	01:03	05:01	02:01	06:03
25	864	01:01	03:01	01:01	05:01	02:01	05:01
26	871	04:03	11:02	03:01	05:05	03:02	03:19
27	871	01:01	12:01	01:01	05:05	03:01	05:01
28	875	01:01	-	01:01	03:03	05:01	-
29	1108	03:01	-	05:01	-	02:01	-
30	1184	04:01	07:01	02:01	03:03	02:02	03:01

Participant	BAU/mL	HLA-DRB1*	HLA-DRB1*	HLA-DQA1*	HLA-DQA1*	HLA-DQB1*	HLA-DQB1*
31	1300	01:01	11:04	01:01	05:01	03:01	05:01
32	1327	11:01	11:02	05:05	-	03:01	03:19
33	1366	03:01	15:01	01:02	05:01	02:01	06:02
34	1545	01:02	13:01	01:01	01:03	05:01	06:03
35	1581	07:01	13:03	02:01	05:05	03:01	03:03
36	1665	04:04	13:02	03:01	-	03:02	-
37	1667	07:01	13:01	01:03	02:01	02:02	06:03
38	1668	07:01	15:01	01:02	02:01	02:05	06:02
39	1716	11:04	13:01	01:03	05:05	03:01	06:03
40	1833	13:01	15:01	01:02	01:03	06:02	06:03
41	1854	03:01	04:01	03:03	05:01	02:01	03:01
42	1865	11:04	15:01	01:03	05:05	03:01	06:01
43	2213	07:01	11:02	02:01	05:05	02:02	03:19
44	2339	07:01	-	02:01	-	02:02	-
45	2382	07:01	15:01	01:02	02:01	02:02	06:02
46	2581	03:01	14:54	01:04	05:01	02:01	05:03
47	3000	04:02	07:01	02:01	03:01	02:02	03:02
48	3128	01:01	03:01	01:03	05:01	02:01	06:03
49	3241	04:04	07:01	02:01	03:01	03:02	03:03
50	3261	01:01	03:01	01:01	05:01	02:01	05:01
51	3650	03:01	11:01	05:01	05:05	02:01	03:01
52	3982	13:02	16:01	01:02	-	05:02	06:04
53	4000	03:01	-	05:01	-	02:01	02:10
54	4014	11:01	15:01	01:03	05:05	03:01	06:01
55	4044	09:01	14:54	01:04	03:02	03:03	05:03
56	4227	04:02	04:04	03:01	-	03:02	-
57	4315	01:02	10:01	01:01	01:05	05:01	-
58	4588	11:01	11:04	03:03	05:05	03:01	03:01
59	4625	03:01	13:01	01:03	05:01	02:01	06:03
60	5122	07:01	15:01	01:02	02:01	02:02	06:02
61	5208	07:01	15:01	01:02	02:01	02:02	06:02
62	5244	03:01	07:01	02:01	05:01	02:01	-

Participant	BAU/mL	HLA-DRB1*	HLA-DRB1*	HLA-DQA1*	HLA-DQA1*	HLA-DQB1*	HLA-DQB1*
63	5419	07:01	-	02:01	-	02:02	03:03
64	5491	13:01	15:01	01:02	01:03	06:02	06:03
65	5521	13:01	15:01	01:02	01:03	06:02	06:03
66	5674	01:03	15:01	01:01	01:02	05:01	06:03
67	5781	11:04	15:01	01:02	05:05	03:01	06:02
68	6152	01:03	15:01	01:01	01:02	05:01	06:02
69	6369	07:01	15:01	01:02	02:01	02:02	06:02
70	6425	03:01	11:01	05:01	05:05	02:01	03:01
71	6727	03:01	04:05	03:03	05:01	02:01	03:02
72	6765	04:01	15:01	01:02	03:01	03:02	06:02
73	6790	10:01	13:02	01:02	01:05	05:01	06:04
74	6958	04:06	07:01	02:01	03:03	02:02	04:02
75	7499	07:01	13:01	01:10	02:01	02:02	06:03
76	7689	04:01	04:04	03:01	03:03	03:01	03:02
77	8109	07:01	13:03	02:01	05:05	02:02	03:01
78	8128	07:01	13:01	01:03	02:01	02:02	06:03
79	8213	07:01	13:02	01:02	02:01	03:03	06:09
80	8423	07:02	13:01	01:03	02:01	03:03	06:03
81	8652	07:01	-	02:01	-	02:02	-
82	8769	04:01	14:54	01:04	03:01	03:02	05:03
83	8951	04:05	-	03:03	-	03:02	-
84	9136	13:02	16:01	01:02	-	05:02	06:09
85	9855	01:01	04:03	01:01	03:01	03:02	05:01
86	10137	11:04	15:01	01:02	05:05	03:01	06:02
87	10505	07:01	-	02:01	02:01	02:02	-

G1 (Low responders group) (<1000 BAU/mL) are marked in blue, G2 (Middle responders) (1000-4400 BAU/mL) in orange and G3 (high responders) (>4400 BAU/mL) in green.
