# Supplementary material for: HLA Class II Polymorphism and Humoral Immunity Induced by the SARS-CoV-2 mRNA-1273 Vaccine
Source: Vaccines (Basel). 2022 Mar 6;10(3):402. doi: 10.3390/vaccines10030402 (PMC8949280; doi:10.3390/vaccines10030402)
Supplement: Supplementary file 1 [file vaccines-10-00402-s001.zip › Supplementary Table S2.pdf]

Supplementary Table S2. HLA allelic frequencies in Vaccinated Population and controls.

Locus	Allele	Controls	Vaccinated Population	Pc
HLA-DRB1	01:01	0.081	0.063	n.s
HLA-DRB1	01:02	0.032	0.046	n.s
HLA-DRB1	01:03	0.009	0.017	n.s
HLA-DRB1	03:01	0.116	0.103	n.s
HLA-DRB1	04:01	0.028	0.029	n.s
HLA-DRB1	04:02	0.019	0.011	n.s
HLA-DRB1	04:03	0.029	0.017	n.s
HLA-DRB1	04:04	0.025	0.040	n.s
HLA-DRB1	04:05	0.012	0.034	n.s
HLA-DRB1	04:06	0.002	0.006	n.s
HLA-DRB1	04:07	0.016	0.011	n.s
HLA-DRB1	07:01	0.164	0.149	n.s
HLA-DRB1	07:02	0	0.006	n.s
HLA-DRB1	08:01	0.022	0.006	n.s
HLA-DRB1	08:02	0.002	0	n.s
HLA-DRB1	08:03	0.002	0	n.s
HLA-DRB1	08:04	0.002	0	n.s
HLA-DRB1	08:06	0.002	0	n.s
HLA-DRB1	09:01	0.006	0.006	n.s
HLA-DRB1	09:02	0.001	0.006	n.s
HLA-DRB1	10:01	0.020	0.011	n.s
HLA-DRB1	11:01	0.068	0.046	n.s
HLA-DRB1	11:02	0.013	0.017	n.s
HLA-DRB1	11:03	0.008	0.011	n.s
HLA-DRB1	11:04	0.031	0.034	n.s
HLA-DRB1	11:33	0.001	0	n.s
HLA-DRB1	12:01	0.010	0.017	n.s
HLA-DRB1	13:01	0.074	0.080	n.s
HLA-DRB1	13:02	0.039	0.040	n.s
HLA-DRB1	13:03	0.017	0.017	n.s
HLA-DRB1	13:05	0.002	0.000	n.s
HLA-DRB1	14:01	0.025	0.006	n.s
HLA-DRB1	14:02	0.001	0	n.s
HLA-DRB1	14:04	0.004	0	n.s
HLA-DRB1	14:35	0.001	0	n.s
HLA-DRB1	14:54	0	0.023	n.s
HLA-DRB1	15:01	0.095	0.115	n.s
HLA-DRB1	15:02	0.013	0	n.s
HLA-DRB1	15:03	0.001	0	n.s
HLA-DRB1	16:01	0.010	0.029	n.s
HLA-DQB1	02:01	0.117	0.103	n.s
HLA-DQB1	02:02	0.138	0.121	n.s
HLA-DQB1	02:05	0	0.006	n.s

HLA-DQB1	02:10	0	0.006	n.s
HLA-DQB1	03:01	0.179	0.155	n.s
HLA-DQB1	03:02	0.092	0.126	n.s
HLA-DQB1	03:03	0.034	0.034	n.s
HLA-DQB1	03:04	0.002	0	n.s
HLA-DQB1	03:05	0.003	0	n.s
HLA-DQB1	03:19	0.001	0.017	n.s
HLA-DQB1	04:02	0.029	0.011	n.s
HLA-DQB1	05:01	0.148	0.121	n.s
HLA-DQB1	05:02	0.016	0.034	n.s
HLA-DQB1	05:03	0.028	0.029	n.s
HLA-DQB1	06:01	0.011	0.011	n.s
HLA-DQB1	06:02	0.091	0.098	n.s
HLA-DQB1	06:03	0.072	0.092	n.s
HLA-DQB1	06:04	0.028	0.017	n.s
HLA-DQB1	06:09	0.009	0.017	n.s

Pc: P corrected by Bonferroni, n.s: Not significant.
