# Supplementary material for: HLA Class II Polymorphism and Humoral Immunity Induced by the SARS-CoV-2 mRNA-1273 Vaccine
Source: Vaccines (Basel). 2022 Mar 6;10(3):402. doi: 10.3390/vaccines10030402 (PMC8949280; doi:10.3390/vaccines10030402)
Supplement: Supplementary file 1 [file vaccines-10-00402-s001.zip › Supplementary Table S3.pdf]

Supplementary Table S3. Haplotypes frequency.

	HLA-DRB1*~HLA-DQA1*~HLA-DQB1*					FREQUENCY			
						G1	G2	G3	ALL CASES
HAPLOTYPES	01:01	~	01:01	~	05:01	0.107	0.034	0.017	0.052
	01:02	~	01:01	~	05:01	0.107	0.034	0	0.046
	03:01	~	05:01	~	02:01	0.071	0.155	0.067	0.098
	04:04	~	03:01	~	03:02	0.054	0.052	0.017	0.040
	04:05	~	03:03	~	03:02	0.054	0	0.050	0.034
	07:01	~	02:01	~	02:02	0.018	0.121	0.200	0.115
	07:01	~	02:01	~	03:03	0	0.034	0.033	0.023
	11:01	~	05:05	~	03:01	0.054	0.052	0.033	0.046
	11:04	~	05:05	~	03:01	0	0.034	0.033	0.023
	13:01	~	01:03	~	06:03	0.054	0.069	0.083	0.069
	14:54	~	01:04	~	05:03	0.018	0.034	0.017	0.023
	15:01	~	01:02	~	06:02	0.054	0.069	0.133	0.086
	16:01	~	01:02	~	05:02	0.054	0.017	0.017	0.029

Haplotypes with more than 2% frequency are represented.
