# Supplementary figures and images for: HLA Class II Polymorphism and Humoral Immunity Induced by the SARS-CoV-2 mRNA-1273 Vaccine
Source: Vaccines (Basel). 2022 Mar 6;10(3):402. doi: 10.3390/vaccines10030402 (PMC8949280; doi:10.3390/vaccines10030402)

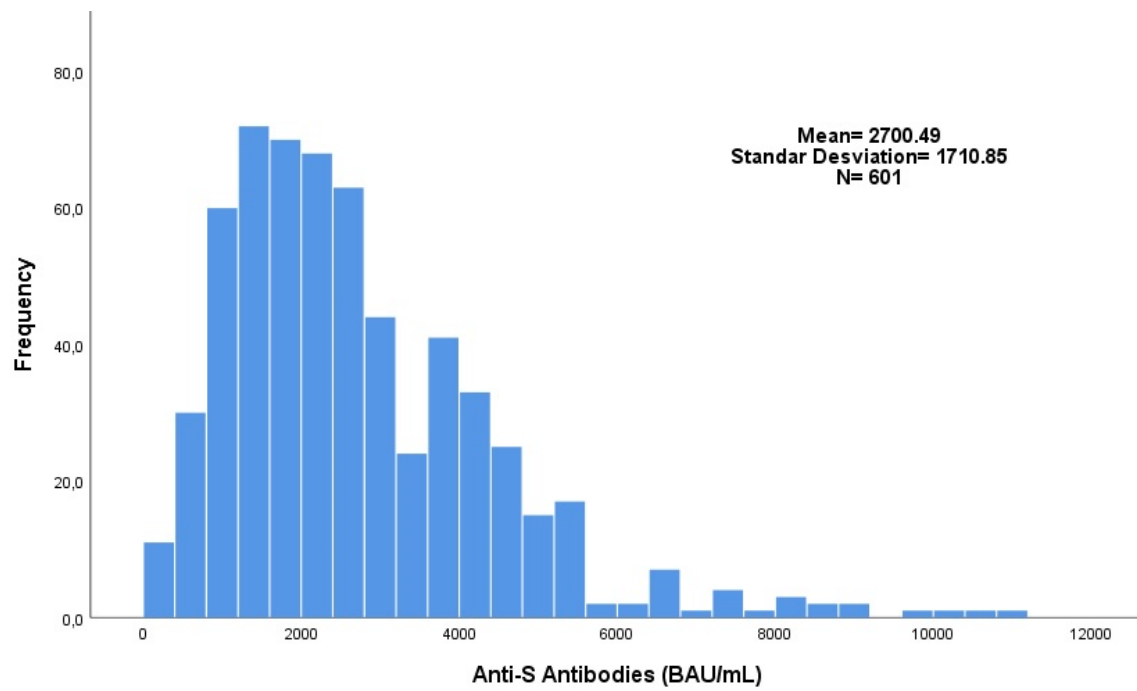

**Figure S1.** Anti-S antibodies groups determination.

Supplement: Supplementary file 1 [file vaccines-10-00402-s001.zip › Supplementary Figure S1.pdf]
